# Assessing angiogenic responses induced by primary human prostate stromal cells in a three-dimensional fibrin matrix assay

**DOI:** 10.18632/oncotarget.11347

**Published:** 2016-08-17

**Authors:** W. Nathaniel Brennen, Huong Nguyen, Susan L. Dalrymple, Stephanie Reppert-Gerber, Jeesun Kim, John T. Isaacs, Hans Hammers

**Affiliations:** ^1^ Department of Oncology, Prostate Cancer Program, The Sidney Kimmel Comprehensive Cancer Center at Johns Hopkins, Johns Hopkins University School of Medicine, Baltimore, MD, USA; ^2^ Present Address: Department of Internal Medicine, Division of Hematology/Oncology, UT Southwestern, Dallas, TX, USA

**Keywords:** angiogenesis, stroma, mesenchymal stem cells (MSC), prostate, stromal paracrine factors

## Abstract

Accurate modeling of angiogenesis *in vitro* is essential for guiding the preclinical development of novel anti-angiogenic agents and treatment strategies. The formation of new blood vessels is a multifactorial and multi-stage process dependent upon paracrine factors produced by stromal cells in the local microenvironment. Mesenchymal stem cells (MSCs) are multipotent cells in adults that can be recruited to sites of inflammation and tissue damage where they aid in wound healing through regenerative, trophic, and immunomodulatory properties. Primary stromal cultures derived from human bone marrow, normal prostate, or prostate cancer tissue are highly enriched in MSCs and stromal progenitors. Using conditioned media from these primary cultures, a robust pro-angiogenic response was observed in a physiologically-relevant three-dimensional fibrin matrix assay. To evaluate the utility of this assay, the allosteric HDAC4 inhibitor tasquinimod and the anti-VEGF monoclonal antibody bevacizumab were used as model compounds with distinct mechanisms of action. While both agents had a profound inhibitory effect on endothelial sprouting, only bevacizumab induced significant regression of established vessels. Additionally, the pro-angiogenic properties of MSCs derived from prostate cancer patients provides further evidence that selective targeting of this population may be of therapeutic benefit.

## INTRODUCTION

Angiogenesis is an essential event in a diverse array of physiologic and pathologic processes, including organogenesis, wound healing, and cancer. Tumors are dependent on angiogenesis to supply needed oxygen and nutrients as they expand and outstrip local resources. Angiogenesis is a tightly controlled process regulated by a series of locally-produced positive and negative factors whose balance dictates vessel formation and stability [1]. The pro- and anti-angiogenic stimuli regulating this process are largely derived from stromal cells in the form of secreted cytokines, growth factors, extracellular matrix components and their proteolytic cleavage products [2–5]. The requirement for stromal-derived paracrine factors in the initiation and maturation of vessels has been demonstrated using primary stromal cultures in a three-dimensional (3D) *in vitro* fibrin matrix co-culture system [2–5]. This assay was originally developed by Hughes et al. and has several advantages over alternative methodologies to assess angiogenic potential in that it accurately recapitulates each of the major physiologic stages necessary for new vessel formation; ultimately resulting in a complex, multicellular capillary network of branched and interconnected lumens [3].

We have previously documented that primary stromal cultures initiated from benign or malignant prostate tissue are highly enriched in mesenchymal stem and/or progenitor cells (MSCs and/or MPCs, respectively; [6]). MSCs are multipotent cells that can functionally differentiate into a variety of mesenchymal lineages, including osteoblasts, adipocytes, chondrocytes, smooth muscle cells, and fibroblasts [6–10]. They are defined analytically based on the co-expression of CD73, CD90, and CD105 in the absence of hematopoietic markers (e.g. CD14, CD20, CD34, CD45, and HLA-DR; [6, 11, 12]). MSCs seem to be present in perivascular niches in tissues throughout the body, but can also be recruited from the bone marrow to sites of tissue damage and inflammation in response to chemokine signals [7, 13–15]. At these sites of damage, MSCs contribute to tissue repair through their regenerative, trophic, and immunomodulatory properties [7, 8, 16]. These properties suggest MSCs play a significant role in promoting wound healing and tissue repair, processes that are closely associated with and dependent upon angiogenesis. Indeed, several studies have demonstrated that MSCs also promote angiogenesis via multiple mechanisms, including the secretion of pro-angiogenic factors (e.g. VEGF, bFGF, and angiopoietin), in addition to expression of proteolytic enzymes (e.g. MMP-2, MMP-9, and MT1-MMP; [17–21]).

Herein, we demonstrate that primary stromal cultures enriched in MSCs and/or MPCs expanded from either human bone marrow, normal prostate, or prostate cancer tissue profoundly induce angiogenesis in a modified version of the previously described 3D *in vitro* assay. Using conditioned media from these cultures, the pro-angiogenic properties were confirmed to be the result of secreted, soluble factors. This experimental setup has the added advantage of being able to evaluate primary cultures associated with high proteolytic activity that are capable of degrading the fibrin matrix. Furthermore, we document this *in vitro* 3D assay represents a robust and tractable methodology to assess the effects of anti-angiogenic agents with different mechanisms of action. For example, the allosteric HDAC4 inhibitor tasquinimod is shown to significantly suppress endothelial sprouting, but has no effect when administered during later stages of angiogenesis. In contrast, the anti-VEGF antibody bevacizumab (Avastin) has a profound inhibitory effect on both sprouting and established vessels, leading to regression of the latter.

## RESULTS

### Defining the critical stages and kinetics of new vessel formation during angiogenesis

**Figure 1:**
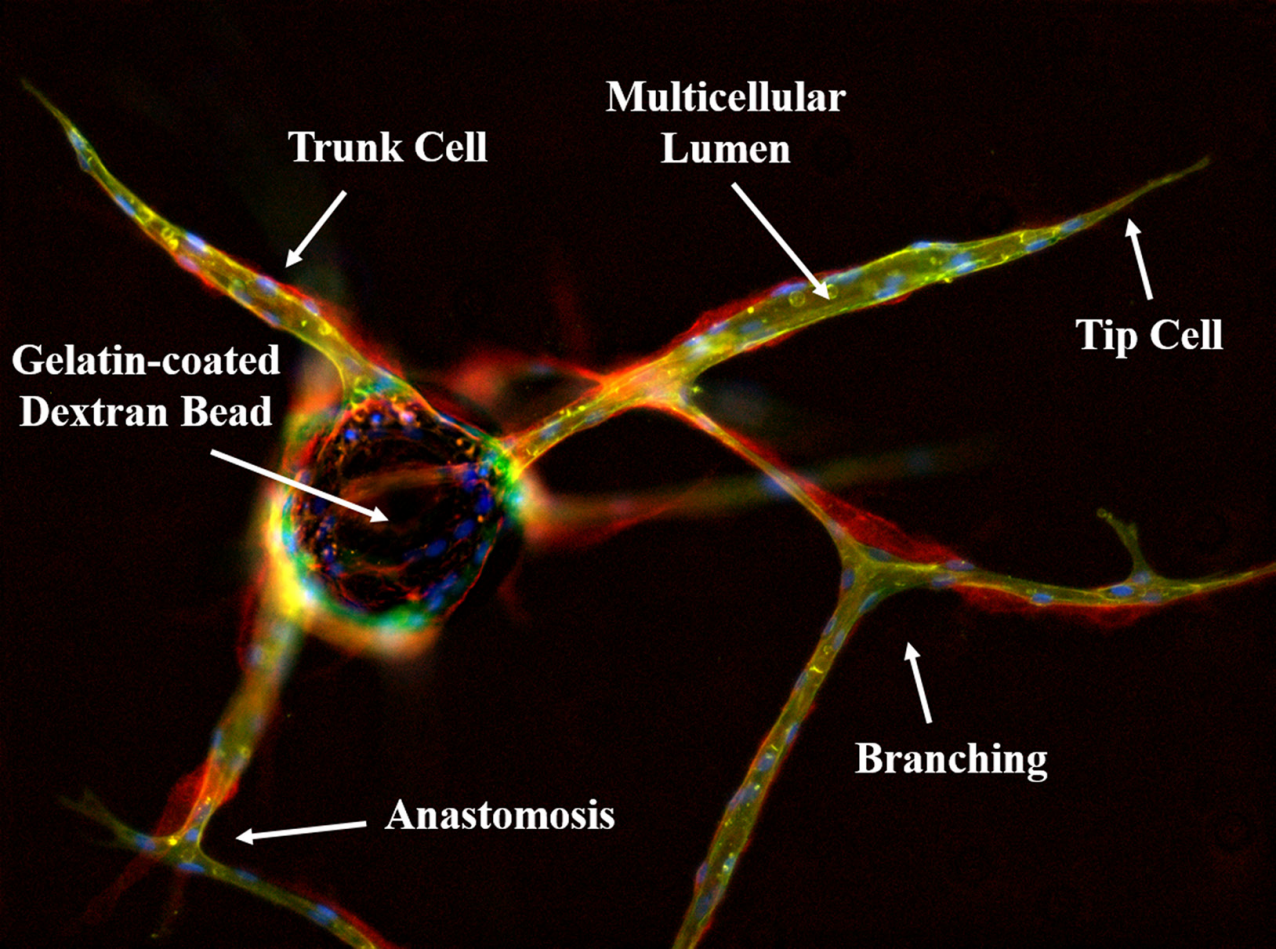
Immunofluorescent characterization of the *in vitro* 3D fibrin matrix assay used to evaluate angiogenesis HUVECs are allowed to adhere overnight to gelatin-coated dextran beads and embedded in a fibrin matrix to simulate the wound healing microenvironment. In the presence of conditioned media from stromal cells, endothelial tip cells invade the surrounding matrix followed by proliferating trunk cells; eventually forming a complex, branched, multicellular capillary network replete with patent lumens. This can be visualized by lectin (green) staining to identify the endothelial cells, which also produce a collagen IV-rich (red) basement membrane. Nuclei are stained blue with DAPI, indicating the multicellular nature of the vessels

Angiogenesis is composed of multiple sequential steps, including sprouting, elongation, branching, and anastomosis. This process is commonly studied in tissue culture using human umbilical vein endothelial cells (HUVECs), including the previously described 3D assay in which cells attached to a gelatin-coated dextran bead are embedded in a fibrin matrix (Figure 1). The use of fibrin is important as this is a physiologically relevant substrate into which endothelial cells would typically invade in the context of angiogenesis and wound healing. Of note, HUVECs embedded in this fibrin matrix on gelatin-coated beads do not sprout under conditions optimized for 2D growth – i.e. media supplemented with VEGF, bFGF, EGF, R3-IGF-1, ascorbic acid, hydrocortisone, heparin, and FBS. Supplementing these cultures with additional exogenous VEGF and bFGF also does not induce sprouting; however, the cells remain attached to the beads and viable. Though VEGF and bFGF are necessary for angiogenesis, these observations clearly demonstrate they are not sufficient. Thus, confirming the absolute dependence of this process on the previously mentioned stromal-derived paracrine factors to be described in greater detail in the discussion.

**Figure 2:**
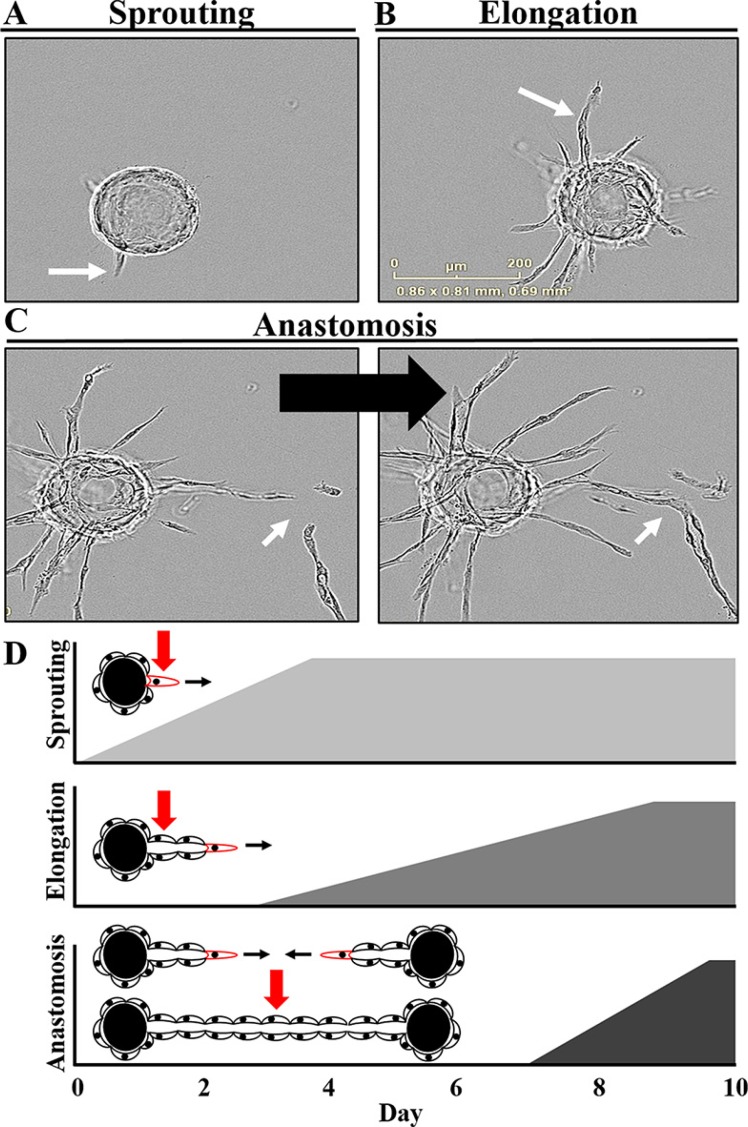
Defining the critical stages and kinetics of angiogenesis *in vitro* (**A**) Sprouting, the initial phase, involves tip cells probing and invading the surrounding fibrin matrix. (**B**) This is followed by the elongation phase in which trunk cells proliferate and migrate to form the growing vessel body behind the invading tip cell. (**C**) A complex, multicellular branched capillary network with patent lumens can then form via anastomosis of proximal vessels. (**D**) The dynamics of this process can be observed over a 10 day assay period with sprouting initiated by the addition of the stromal-derived factors. Once these sprouts begin to stabilize over the first few days, the elongation phase begins, continuing for the remainder of the assay. Lumen formation can be detected on approximately day 4–5 with branching and anastomosis of proximal vessels taking place over the final days of the assay. Also see Supplemental Movie S1.

Following addition of the required stromal factors via co-culture or conditioned media, sprouting and vessel formation can be observed *in vitro* over a 10 day assay period (Figure 2, Supplementary Movie S1). Over the first few days, multiple individual tip cells rapidly penetrate the surrounding fibrin matrix (Figure 2A, 2D). Several rounds of lamellipodia and filapodia extension are observed prior to sprout stabilization beginning on approximately day 2. This is followed by the elongation phase during which trunk cells proliferate and migrate over the remainder of the assay to form the growing vessel body behind the invading tip cell (Figure 2B, 2D). Lumen formation can be detected on approximately day 4–5 with branching beginning around day 6–7. Subsequently, proximal vessels can undergo anastomosis to form a capillary network of interconnected lumens (Figure 2C, 2D).

**Figure 3:**
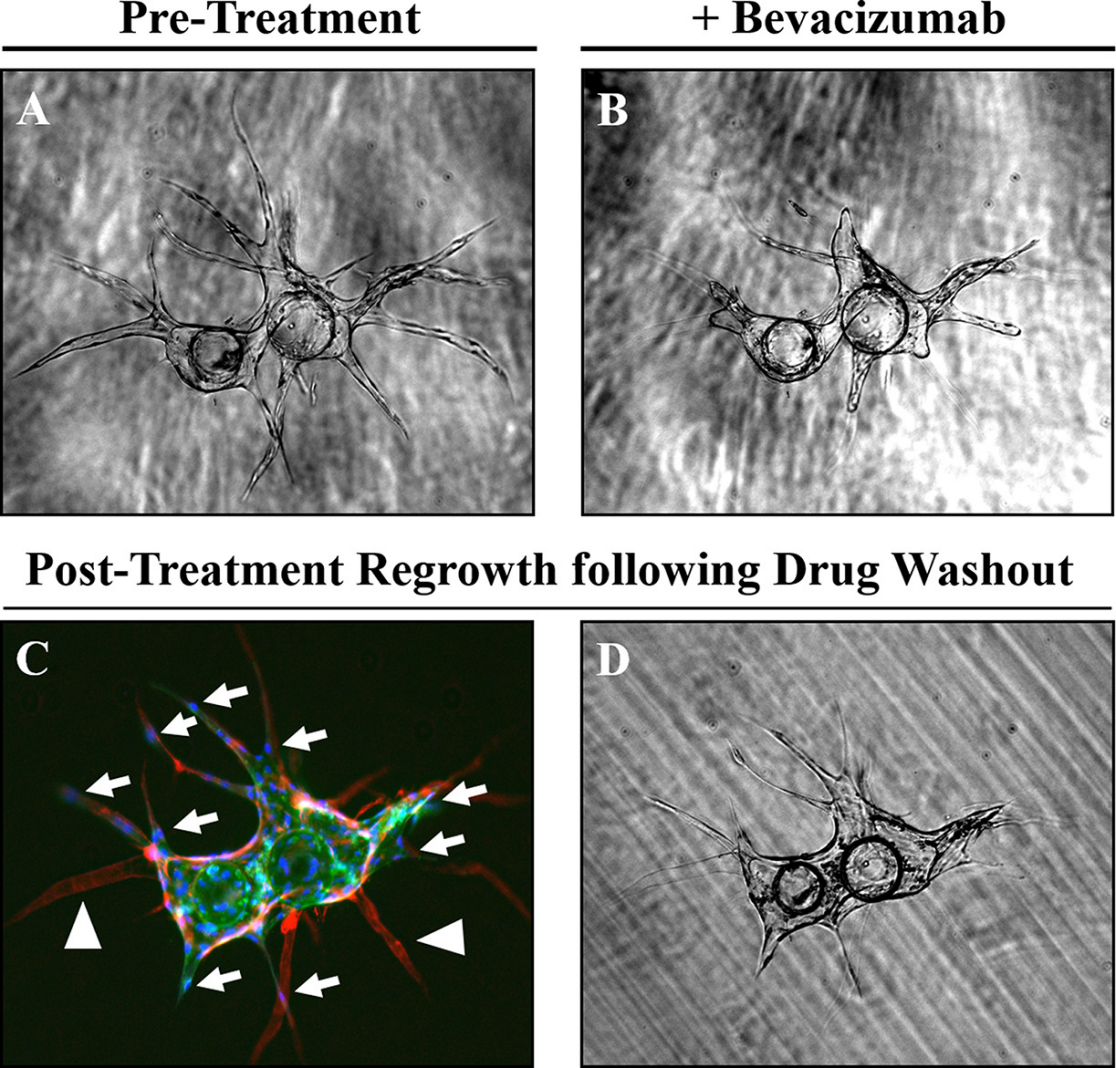
Regression and regrowth of established vessels along pre-defined basement membrane tracts (**A**) Regression of established vessels can be induced using an anti-VEGF treatment. (**B**) such as bevacizumab (10 μg/mL). (**C**) Regression of these vessels [lectin-positive, (green)] leaves behind an acellular [DAPI-negative, (blue)] basement membrane ‘tunnel’ defined by collagen IV (red) staining and indicated by the white arrowheads. Following drug wash out, tip cells indicated by white arrows can migrate along this basement membrane network to re-establish the pre-existing vessel network (**D**).

Importantly, these endothelial vessels [lectin-positive (green)] produce a collagen IV-rich basement membrane [red, (Figure 1)]. Regression of these established vessels can subsequently be induced with an anti-VEGF agent, such as bevacizumab (Figure 3A, 3B); leaving behind an acellular, collagen-rich ‘sleeve’ or tunnel that serves as a track for vessel regrowth following removal of the anti-angiogenic agent [arrowhead, (Figure 3C)]. This is demonstrated by the migration of tip cells along these same basement membrane networks during the initial phases of vessel regrowth following drug washout [arrows, (Figure 3C)]; ultimately recapitulating the pre-existing capillary network to a large extent (Figure 3D). These observations demonstrate this is a robust model that can be used to follow dynamic responses to pro- and anti-angiogenic factors, including pharmaceutical agents, in real time. Further, these data indicate that although angiogenesis is a multifactorial process dependent upon multiple inputs, only one of these ‘necessary but not sufficient’ pathways needs to be targeted to produce a significant anti-angiogenic effect.

### Primary human prostate stromal cultures enriched in MSCs and/or MPCs potently induce angiogenesis

Traditionally, normal human lung fibroblasts (NHLFs) are used as the source of stromal-derived pro-angiogenic factors in this assay [2–5]. To determine whether human prostate stromal cells can also induce angiogenesis in this assay, primary human prostate stromal cultures were initiated from young organ donors (< 25 yo) and radical prostatectomy patients, which represent benign and malignant tissue, respectively. Comparable primary prostate stromal cultures were previously shown to be enriched in MSCs and MPCs [6, 12]. Thus, their angiogenic potential was compared to that of canonical bone marrow-derived MSCs, in addition to utilizing NHLF cultures as positive controls to be consistent with established precedent.

The multilineage differentiation potential of these primary stromal cultures was confirmed in addition to quantifying the percentage of MSCs present in each culture based on the previously described analytical markers using an optimized multiparameter flow cytometry assay [6, 12]. Based on this assay, the majority of cells (> 60%) in all cultures were consistent with an MSC phenotype based on surface marker expression at the time of analysis [passage ≥ 3, (Table 1)]. Consistent with previous observations [6, 12], stromal cells expanded from normal prostate tissue (i.e. nPrSCs) could generate osteoblasts and chondrocytes, but were unable to differentiate into adipocytes. Therefore, primary prostate stromal cells from young organ donors represent lineage-restricted MPCs as opposed to MSCs, which are characterized by a broader differentiation potential. In contrast and similar to bone marrow-derived MSCs, a subset of stromal cultures initiated from radical prostatectomy tissue (i.e. PrCSCs) retain their adipogenic differentiation potential (Table 1). Of note, NHLFs were also consistent with an MSC phenotype based on these analytical markers. However, like the nPrSCs, NHLFs were unable to differentiate into adipocytes, though they retained the ability to generate osteoblasts and chondrocytes (Table 1), suggested they also represent lineage-restricted MPCs.

**Table 1:** Characterization of multipotent differentiation potential and MSC phenotypic markers in primary human stromal cultures derived from multiple tissue sources

Tissue Source	Sample	Donor Age (yrs)	Gleason Score	Adipocyte	Osteoblast	Chondrocyte	MSCs (%)
**Bone Marrow**	**BM-MSC-1**	25	N/A	+	+	+	+
	**BM-MSC-2**	25	N/A	+	+	+	+
**Prostate Cancer**	**PrCSC-1**	47	3 + 3	+	+	+	+
	**PrCSC-2**	59	3 + 4	-	+	+	+
**Normal Prostate**	**nPrSC-1**	24	N/A	-	+	+	+
	**nPrSC-2**	25	N/A	-	+	+	+
**Normal Lung**	**NHLF**		N/A	-	+	+	+

Adipogenesis was scored based on positive Oil Red O staining. Osteogenesis was scored based on positive Alizaren Red S staining. Chondrogenesis was scored based on positive Alcian Blue staining. The percentage of MSCs present in a culture was defined using flow cytometry as previously described (10, 16) based upon ≥ 60% of the population being CD73^+^/CD90^+^/CD105^+^ triple-positive in the absence of hematopoietic lineage markers (CD14^−^, CD20^−^, CD34^−^, CD45^−^, and HLA-DR-).

Initially, cells from these primary stromal cultures were overlaid on the fibrin matrix to assess their angiogenic potential according to previously described methods [3]. However, this frequently resulted in degradation of the matrix due to the high proteolytic activity present in some of the cultures tested as evidenced by the presence of cells along the bottom of the tissue culture well following matrix collapse (Supplementary Figure S1). It should be noted that despite matrix degradation, a pro-angiogenic response was typically still observed. This further supports the role of MSCs and/or MPCs in promoting tissue repair in a physiologically relevant setting that consists of a complex extracellular matrix with multiple different structural proteins, which are needed to provide mechanical support as the fibrin matrix is degraded during repair. However, fibrinolysis not only made the assay more variable, but also made accurate assessment difficult or impossible in many cases.

**Figure 4:**
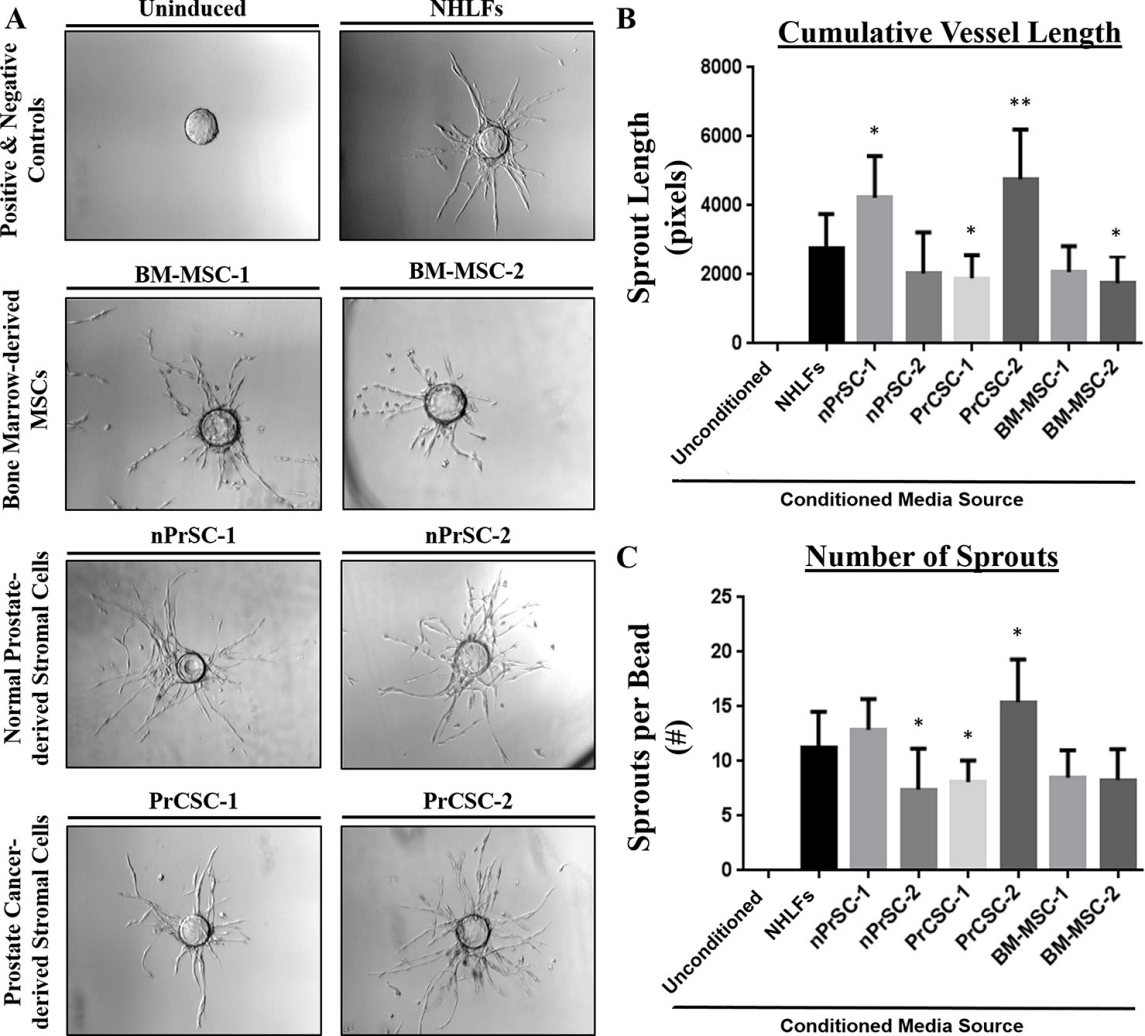
Primary human prostate stromal cultures enriched in MSCs and/or MPCs potently induce angiogenesis (**A**) Conditioned media from primary stromal cultures derived from benign and malignant prostate tissue (nPrSCs and PrCSCs, respectively) induce robust vessel formation comparable to or surpassing that achieved using bone marrow-derived MSCs and normal human lung fibroblasts (NHLFs), the positive control in this assay. The uninduced negative control includes fully supplemented EGM2 media, but no stromal conditioned media was added. This response was quantified by measuring (**B**) cumulative vessel length and (**C**) the number of sprouts per bead. Importantly, conditioned media from prostate cancer cell lines (CWR22Rv1, LNCaP, and PC3) were also unable to induce sprouting in this assay (i.e. sprout length < 100 pixels, sprouts per bead < 1). All assays performed in triplicate. Ten beads analyzed per condition. All statistical comparisons made relative to NHLFs. *p < 0.05. **p < 0.005.

Therefore, to standardize the assay and reduce variability, concentrated conditioned media was utilized in all subsequent experiments to assess the angiogenic potential of paracrine factors secreted from each of these cultures. As shown in Figure 4A, conditioned media from each of these primary cultures was able to induce a robust pro-angiogenic response equivalent to or even surpassing that of the NHLFs with regards to cumulative vessel length (Figure 4B) and sprout number (Figure 4C). Though all stromal cultures tested were able to promote sprouting, it should be noted this is not a non-specific response as demonstrated by the lack of sprouting (sprout length < 100 pixels and sprouts per bead < 1) observed using conditioned media from multiple prostate cancer cell lines (CWR22Rv1, LNCaP, and PC3).

### Targeting distinct phases of the angiogenic response with anti-angiogenic agents

**Figure 5:**
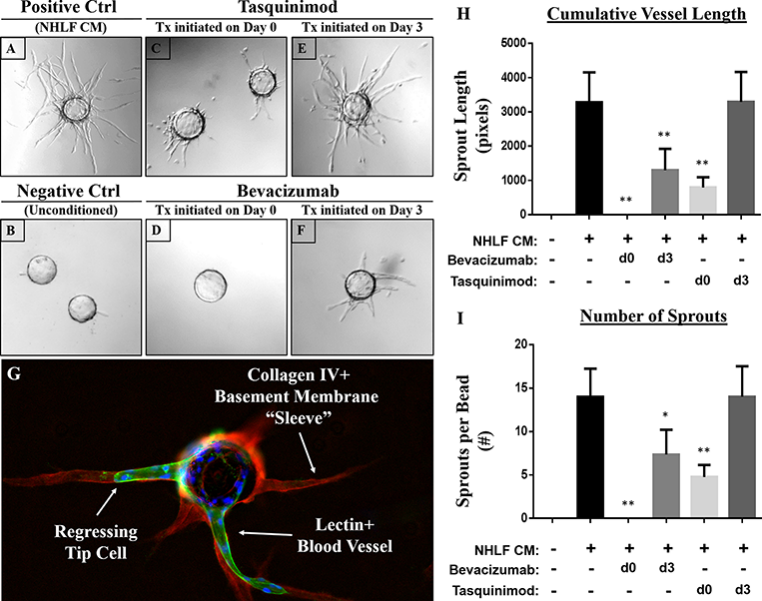
Targeting distinct phases of the angiogenic response using anti-angiogenic agents with distinct mechanisms of action (**A**) The addition of conditioned media from NHLFs represents the positive control, while cultures containing fully supplemented EGM2 but no stromal-conditioned media represents the (**B**) negative control. Tasquinimod (10 μM), an allosteric inhibitor of HDAC4, significantly suppresses vessel sprouting when added upon assay initiation (**C**) but not when given on day 3 once the vessels have sprouted (**E**) Like tasquinimod, the anti-VEGF monoclonal antibody bevacizumab (10 μg/mL) profoundly inhibits sprouting when administered on day 0 (**D**) in addition to inducing a significant regression of established vessels when added on day 3 (**F**) as indicated by the presence of an acellular [lectin (green) and DAPI (blue) -negative] basement membrane sleeve defined by collagen IV (red) staining (**G**) Quantification of these responses was performed by measuring (**H**) cumulative vessel length and (**I**) the number of sprouts per bead. Assays performed in triplicate. Ten beads analyzed per condition. All statistical comparisons made relative to NHLFs. *p < 0.001. **p < 0.0001.

To determine whether this assay could be utilized to determine which stage of the angiogenic response was being targeted by novel anti-angiogenic compounds, we utilized two drugs with known anti-angiogenic properties and distinct mechanisms of action. Tasquinimod is an orally-available allosteric inhibitor of HDAC4 that blocks its interaction with HIF-1α and consequently, downstream pro-angiogenic responses with an IC50 of ~0.5–1 μM and maximal responses achieved at 10 μM [22]. When administered upon assay initiation (i.e. day 0), tasquinimod (10 μM) clearly suppresses angiogenesis as demonstrated by a reduction in overall sprout number and length relative to untreated controls (Figure 5C). In contrast, no effect is observed on established vessels if they are allowed to sprout prior to administering tasquinimod on day 3 (Figure 5E).

Like tasquinimod, bevacizumab (10 μg/mL), an anti-VEGF monoclonal antibody, significantly suppresses endothelial sprouting when administered on day 0 of the assay (Figure 5D). Unlike tasquinimod, however, it also induces significant vessel regression when applied during later stages (i.e. day 3) of the assay (Figure 5F). This is clearly demonstrated by the empty ‘tunnel’ left behind following regression that is demarcated by an acellular, collagen type IV-rich (red) basement membrane ‘sleeve’ (Figure 5G). A representative image of untreated cells is shown in Figure 1 for comparison. Quantification of these responses (i.e. cumulative vessel length and number of sprouts) are presented in Figure 5H and 5I, respectively. These observations suggest that bevacizumab is able to target both sprouting and established vessels; whereas, tasquinimod only has an effect during the initial stages of vessel development.

## DISCUSSION

Several preclinical assays have been developed to study angiogenesis, including endothelial tube formation, chick chorioallantoic membrane (CAM), and aortic ring assays. Though commonly used, each of these assays has significant limitations that prevent robust and practical evaluation of novel compounds or fail to accurately model the angiogenic process in humans. For example, the matrigel tube or cord formation assay is frequently used as the gold standard *in vitro* angiogenesis assay. However, cord-like morphogenesis can not only be observed with non-endothelial cells [23], but was also found to be independent of transcription and translation [24]; questioning the ability of this assay to accurately reflect the complex biological processes associated with true angiogenesis. To overcome these limitations, Hughes et al. developed an *in vitro* 3D assay utilizing HUVECs attached to gelatin-coated dextran beads and embedded in a fibrin matrix [2–5].

Herein, we also demonstrate this is a robust assay for dissecting the effects of anti-angiogenic agents that may be predictive of anti-tumor responses *in vivo*. As examples, bevacizumab and tasquinimod were selected as model compounds with distinct mechanisms of action. Though both agents were able to suppress sprouting during the initial stages of vessel development, only bevacizumab was able to induce significant regression of established vessels. Bevacizumab is FDA-approved for clinical use in advanced stages of cervical, colorectal, lung, renal, and brain cancers. These approvals were largely based on increases in overall (OS) and progression-free survival (PFS) when used in combination with effective multi-drug cytotoxic chemotherapy. In contrast, bevacizumab alone or in combination with single agent taxane-based chemotherapy has not proven effective in prostate cancer [25, 26]. As pointed out, however, bevacizumab was discontinued in the phase III trial at the time of PSA or radiographic progression resulting in a shorter duration of treatment, which may have compromised potential therapeutic benefits [25, 26]. This is supported by the quickly reversible effects of anti-VEGF therapy demonstrated herein by rapid vessel regrowth following drug washout, suggesting extended chronic exposure to bevacizumab is necessary to achieve sustained therapeutic effects. Tasquinimod is an investigational drug being evaluated in metastatic castration-resistant prostate cancer (mCRPC) and other solid tumor types. Unfortunately, tasquinimod failed to increase OS when given at 1 mg/day in a recent phase III trial of men with chemotherapy-naïve mCRPC, but did significantly improve radiographic PFS in this population [27]. It should be noted that this dose achieves a C_max_ of ~0.5 μM, which is suboptimal based on an IC50 for this agent of ~0.5–1 μM [22]. As demonstrated, maximal responses are achieved at a dose of 10 μM, suggesting the patients in this trial were significantly under-dosed.

A dependence upon stromal-derived factors has been strongly documented in multiple stages of angiogenesis, including sprouting and lumen formation [2–5]. Hepatocyte growth factor (HGF) and fibronectin are particularly important for promoting endothelial sprouting; though angiopoietin-1 (ANG-1), angiogenin, transforming growth factor (TGF)-α, and tumor necrosis factor (TNF) have also been implicated in this stage of vessel development [4]. Interestingly, these factors do not have a significant effect on lumen formation, a process requiring a distinct constellation of stimuli, including collagen type I alpha-1 (Col1A1), procollagen C-endopeptidase enhancer 1 (PCOLCE), transforming growth factor-β-induced protein ig-h3 (βig-h3), insulin-like growth factor-binding protein 7 (IGFBP7), and secreted protein acidic and rich in cysteine (SPARC, [5]). These stromal-derived paracrine factors are typically provided using NHLFs or other primary stromal cultures frequently referred to as fibroblasts.

Of note, primary stromal cultures initiated from benign or malignant human prostate tissue are rapidly enriched in cells that are functionally and analytically consistent with a mesenchymal stem or progenitor cell phenotype (MSCs and MPCs, respectively, [6]). Though the NHLFs traditionally used in this assay are unable to differentiate into adipocytes, they are consistent with other canonical MSC properties; indicative of a more lineage-restricted MPC phenotype similar to that observed in primary stromal cultures derived from normal prostate tissue [6]. Thus, commercially-available ‘fibroblast’ cultures are also enriched in MSCs and/or MPCs; an important point when considering the source of the previously highlighted stromal-derived pro-angiogenic factors.

MSCs are present in all tissues throughout the body at relatively low levels [7, 13, 14], including the prostate [6, 12], suggesting they may act as a local sensor of tissue damage and promote the overall repair process. Importantly, MSCs can also be mobilized from the bone marrow and recruited to sites of tissue damage in response to chemokine signals, including CXCL12, CCL5, and CCL2 [7, 15]. At these lesions, MSCs contribute to the repair process via immunomodulatory properties to suppress fibrosis and chronic inflammation, trophic properties to promote growth and survival, and their multipotent differentiation potential to regenerate tissue-specific stromal elements [7–10, 16]. As demonstrated herein, MSCs also contribute to wound healing through inducing angiogenesis via paracrine factors.

Given the fact that tumors are frequently described as ‘wounds that do not heal’ and the documented association between inflammation and prostate cancer [7, 28–30], it is unsurprising that MSCs can also be identified in human prostate cancer tissue [12]. MSC infiltration from the bone marrow in response to tumor progression is further supported by the observation that less committed progenitors or MSCs are detected in radical prostatectomy tissue from a subset of men with prostate cancer; whereas, more lineage-restricted progenitors or MPCs are typically present in normal tissue [6, 7]. The observation that primary stromal cultures from normal lung tissue (i.e. NHLFs) are also consistent with an MPC phenotype, suggests this may represent an emerging pattern in which contextual, tissue-specific restriction of differential potential is observed in stromal progenitors expanded from normal tissues; whereas, MSCs with a broader differentiation repertoire are only observed in high numbers in areas of tissue damage or under pathological conditions. Though bone marrow-derived MSCs have previously been associated with pro-angiogenic properties [7, 17–21], this is the first report of MSCs and MPCs specifically isolated from normal and malignant prostate tissue having this same potential. This provides additional evidence that selective targeting of this population may be of therapeutic benefit. Furthermore, the recruitment of these cells to the prostate suggests they can potentially be exploited for the delivery of cytotoxic or imaging agents [31].

In summary, the previously reported 3D *in vitro* fibrin matrix assay further characterized herein represents a robust and practical methodology for evaluating the angiogenic potential of multiple stromal cell populations derived from primary tissue sources. Though fibroblasts are traditionally used in this assay, increasing evidence suggests these cultures are frequently enriched in cells consistent with a stem or progenitor phenotype (i.e. MSCs and MPCs); a point of general importance when interpreting published data. Primary stromal cultures enriched in MSCs and MPCs initiated from benign or malignant prostate tissue were shown to induce robust sprouting and vessel formation. The pro-angiogenic properties of MSCs derived from prostate cancer patients provides further evidence that selective targeting of this population may be of therapeutic benefit. Additionally, we demonstrated the anti-angiogenic effects of novel agents on distinct phases of angiogenesis can be interrogated utilizing this assay, providing data that may inform further clinical development or implementation of existing agents.

## MATERIALS AND METHODS

### Reagents

RPMI Medium 1640 and 0.25% trypsin-EDTA were from Invitrogen (Grand Island, NY). EBM2 Medium supplemented with VEGF, bFGF, epidermal growth factor (EGF), R3-insulin-like growth factor-1 (R3-IGF-1), ascorbic acid, hydrocortisone, heparin, fetal bovine serum (FBS) and penicillin/streptomycin (i.e. EGM2). All of which were from Lonza (Walkerside, MD) with the exception of FBS, which was obtained from Gemini Bio-Products (West Sacramento, Ca). Cytodex beads were from Amersham Biosciences (Uppsala, Sweden). Gelatin Type A was from MP Biomedicals (Aurora, OH). 0.22 μm pore-size syringe filter, 0.22 μm pore-size bottle-top filter, and 50 kDa Centrifugal filter was from Millipore (Cork, Ireland). Tasquinimod was provided by ActiveBiotech (Lund, Sweden). Commercially available bevacizumab was obtained from the Johns Hopkins pharmacy.

### Primary tissue and cell culture

Tissue was collected in accordance with Johns Hopkins Institutional Review Board (IRB)-approved protocols. Bone marrow-derived MSCs (BM-MSC) were cultured from healthy bone marrow donor aspirates as previously described [6, 12] or purchased from RoosterBio (Frederick, MD); one of each were used (*n* = 2 total). Normal prostate tissue was obtained from young organ donors < 25 years of age (*n* = 2) through the National Disease Research Interchange (NDRI) as previously described [6]. Prostate Cancer tissue was harvested from men undergoing radical prostatectomy (*n* = 2) at the Brady Urological Institute at Johns Hopkins also as previously described [6].

Tissue was mechanically and enzymatically digested into a single cell suspension according to previously optimized protocols [6, 12] using a human tumor dissociation kit (Miltenyi Biotec, Inc. Bergisch Gladbach, Germany) and gentleMACS dissociator (Miltenyi) in accordance with the manufacturer's instructions. The single cell suspension was washed in FBS-supplemented media prior to plating for subsequent culturing in RoosterBio High Performance Media or RPMI 1640 supplemented with 10% FBS as previously described [6, 12].

HUVECs, normal human lung fibroblasts (NHLF), and DU145 were purchased from ATCC. The CWR22-Rv1 cell line was obtained as previously described [32]. HUVECs were cultured in fully supplemented EGM2 Medium and all other cells were grown in RPMI 1640 with 10% FBS and 1% penicillin/streptomycin. All cells were maintained at 37°C in 5% CO_2_ and 95% air incubator. All cell lines were routinely tested for mycoplasma contamination using the MycoSensor PCR Assay kit (Agilent Technologies, Santa Clara, CA) and authenticated using STR analysis by the Johns Hopkins Genetic Resources Core Facility.

### Quantification and differentiation of MSCs

Multiparameter flow cytometry was used to quantify MSCs in primary cultures based on the co-expression of CD73, CD90, and CD105 in the absence of CD14, CD20, CD34, CD45, and HLA-DR as previously described [6, 12]. MSC differentiation assays were performed using adipogenic, osteogenic, and chondrogenic differentiation reagents from Lonza according to manufacturer's instructions [6, 12].

### Conditioning media

All cells were passaged and maintained to confluent in T175 Flask with 25mL of RPMI supplemented with 10% FBS and 1% penicillin/streptomycin. Media was collected every 2–3 days from confluent flasks and filter-sterilized with a 0.22 μm pore-size bottle-top filter and stored at 4°C. A total of 100 mL of collected media for each cell type was concentrated and washed with PBS using a 50 kDa centrifugal filter to 1.5 mL.

### Sprouting assay

On day 1, 500 μL of 50 ml/g Cytodex beads were coated with 3 mL of 0.5% gelatin Type A solution (0.5% gelatin type A in PBS and 1% penicillin/streptomycin) in a 15 mL tube for 1 hr at 37°C in 5% CO_2_ and 95% air incubator. Three milliliters of Gelatin Type A solution was removed and 2 mL of EGM2 Medium added to the same tube containing the cytodex beads. The cap was loosely tightened to equilibrate EGM2 Medium for 30 min in a 37°C tissue culture incubator. HUVEC cells (1.5 × 10^6^) were suspended in 1 mL of EGM2 Medium and added to the equilibrated beads. The tube was positioned on its side and mixed every 20 min to allow for maximal attachment of HUVEC cells to the gelatin-coated cytodex beads. After 4 hr, all of the HUVEC-coated beads (HUVEC:beads) were added to 10 mL of EGM2 Medium and 1 mL of FBS in a T25 flask and placed in a 37°C incubator overnight to allow for cell spreading on the beads and removal of residual unattached cells.

On day 2, a fibrinogen solution was prepared by slowly mixing 60 mg of fibrinogen in 20 mL of PBS for 2 hr in a 50 mL conical tube. One milliliter of FBS was then added to the fibrinogen solution and sterile-filtered using a 0.22 μm pore-size syringe filter. The HUVEC:bead mixture (10 mL) in the T25 flask from the previous day were transferred into a 15 mL conical tube. The HUVEC:beads were allowed to settle to the bottom of the tube, then 9 mL of the remaining EGM-2 Medium was removed. HUVEC:beads were washed with 3 mL of PBS, and again allowed to settle to the bottom of the tube before aspirating the remaining PBS. HUVEC:beads were resuspended with 4 mL of the fibrinogen solution and transferred to the original fibrinogen solution in the 50 mL conical tube, creating a HUVEC:beads:fibrinogen mixture.

Thrombin (50 U) was dissolved in 3 mL of PBS. The thrombin solution (7.9 μL or 30 μL, respectively) was pipetted into a 96- or 48-well non-tissue culture-treated plate. The HUVEC:beads:fibrinogen mixture (60 μL or 200 μL, respectively) was added on top of the thrombin solution in the 96- or 48-well non-tissue culture-treated plate by pipetting. The HUVEC:beads:fibrinogen and thrombin matrix was allowed to stabilize for 5 min at room temperature before placing it into a 37°C incubator for 1 hr before the addition of conditioned media prepared as described above. In 96-well plates, 180 μL of EGM2 Medium and 50 μL of conditioned media were added on top of the HUVEC:beads:fibrinogen and thrombin matrix. In 48-well plates, 20,000 cells suspended in 800 μL of EGM-2 and added on top of the HUVEC:beads:fibrinogen and thrombin matrix. Assays were run in triplicate. Images of individual beads were taken on days 5 and 11.

### Image analysis

Images of individual beads were taken under a fluorescence microscope at 60 x magnification. The length and number of sprouts were measured via computer-assisted image analysis using the public domain program, ImageJ. Lumenized sprouts were measured using the free-hand tool starting at the edge of the bead to the tip of the sprout. Then each measurement was labeled numerically. Short and unlumenized sprouts consisting of a single HUVEC cell were not measured. The cumulative sprout length per bead was recorded and averaged for each cell type. The number of sprouts per beads were also recorded and averaged for each cell type. Sprouts from 10 beads quantified per condition.

### Immunofluorescence

Endothelial cell surface, basement membrane, and nuclei stained with FITC-labeled Ulex europaeus lectin (Vector labs), mouse anti-collagen IV [COL-94, (Sigma)]/anti-mouse Alexa 594 antibody, and DAPI respectively. Pictures were obtained on a Nikon TE200 fluorescence microscope using the Metamorph software package (Universal Imaging) and were process in ImageJ software.

### Statistical analysis

Statistical analysis was done using either Student's *t*-test or ANOVA to compare multiple groups. All groups were compared to NHLF. *P* < 0.05 was considered statistically significant.

## SUPPLEMENTARY MATERIALS FIGURES AND MOVIES






