# Supplementary material for: Assessing angiogenic responses induced by primary human prostate stromal cells in a three-dimensional fibrin matrix assay
Source: Oncotarget. 2016 Aug 17;7(44):71298–308. doi: 10.18632/oncotarget.11347 (PMC5342079; doi:10.18632/oncotarget.11347)
Supplement: Supplementary file 1 [file oncotarget-07-71298-s001.pdf]

Assessing angiogenic responses induced by primary human prostate stromal cells in a three-dimensional fibrin matrix assay

Supplementary Materials

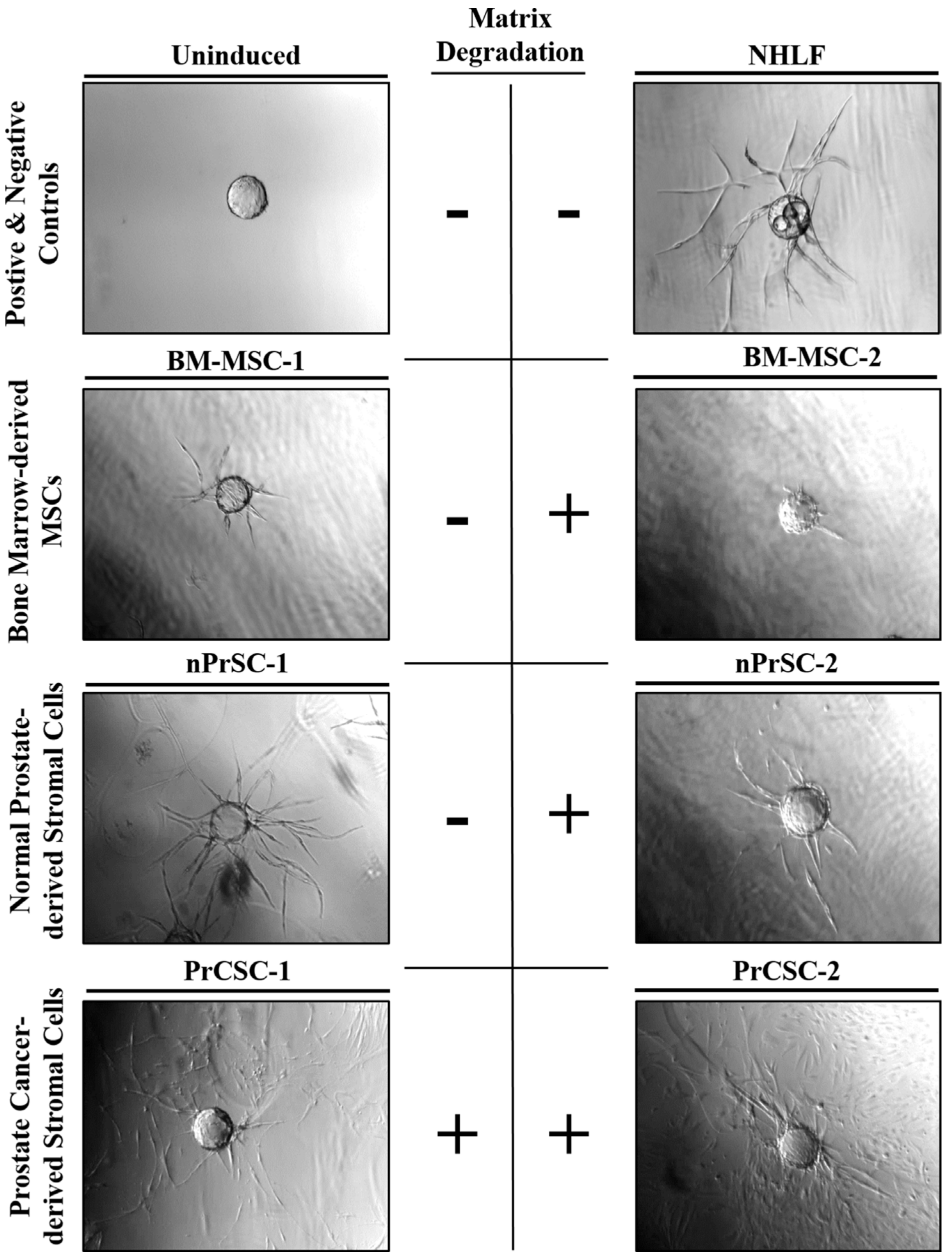

Supplementary Figure S1: Stromal cells with high proteolytic activity can lead to significant inter-assay variability, making accurate assessment difficult as a result of matrix degradation when the cells are plated directly on top of the fibrin matrix in a co-culture setup for direct conditioning of the assay media. Therefore, using concentrated conditioned media from an independent stromal culture that can be added to the assay media is recommended for standardization of the assay and to reduce variability.

Supplementary Movie S1: Kinetics of vessel formation induced using concentrated conditioned media from stromal cells in the 3D *in vitro* fibrin matrix assay over 10 days. See Supplementary_ Movie _S1.
